# Is Extended Lymphadenectomy in Biliary Tract Cancers Justified? A Retrospective Comparative Study of Gallbladder Cancer, Perihilar and Intrahepatic Cholangiocarcinoma

**DOI:** 10.1245/s10434-026-19192-1

**Published:** 2026-02-16

**Authors:** Esther Giehl-Brown, Rajan Nikbakhsh, Sarah Bendig, Olga Radulova-Mauersberger, Johannes Schweipert, Jürgen Weitz, Carina Riediger

**Affiliations:** 1 Department of Visceral, Thoracic, and Vascular Surgery, University Hospital Carl Gustav Carus, Technische Universität Dresden, Dresden, Germany; 2 National Center for Tumor Diseases/University Cancer Center, Dresden, Germany; 3 Faculty of Medicine, University Hospital Carl Gustav Carus, Technische Universität Dresden, Dresden, Germany; 4 Department of General, Visceral, and Transplantation Surgery, University of Heidelberg, Heidelberg, Germany; 5 Department of General, Visceral, and Thoracic Surgery, Marienhospital Stuttgart, Stuttgart, Germany

**Keywords:** Biliary tract cancer, Lymphadenectomy, Staging accuracy, Recurrence-free survival, Postoperative morbidity

## Abstract

**Background:**

The oncologic value of lymphadenectomy (LND) in biliary tract cancers (BTC) remains controversial. While guidelines recommend retrieval of ≥ 6 lymph nodes to ensure accurate staging, evidence for a therapeutic survival benefit is limited.

**Patients and Methods:**

We retrospectively analyzed 253 consecutive resections for intrahepatic cholangiocarcinoma (iCCA), perihilar cholangiocarcinoma (pCCA), and gallbladder carcinoma (GBC) at a high‑volume hepatobiliary center from a prospectively maintained database (2013–2023). Patients were stratified into no (0 nodes), limited (1–5 nodes), and extended (≥ 6 nodes) LND. Postoperative morbidity, recurrence-free survival (RFS), and overall survival (OS) were assessed with uni- and multivariable models.

**Results:**

LND was performed in 47% of patients and extended LND in 52.9%. Clavien–Dindo grade ≥ III complications occurred in 69.8% with LND ≥ 6 compared with 46.4% with LND1–5 and 41.8% with no LND (*p* < 0.001), with longer ICU and hospital stays and more septic and pulmonary events. On multivariable analysis, LND ≥ 6 was not an independent predictor of morbidity in the overall cohort, but in the subgroup of major resections (OR 2.79, 95% CI 1.121–6.955, *p* = 0.027). LND extent had no independent impact on OS or RFS.

**Conclusions:**

Extended LND was associated with a higher rate of postoperative complications and was an independent risk factor in patients undergoing major hepatectomy. However, no clear survival benefit was observed. These findings may suggest that the role of LND in BTC may be primarily diagnostic and that more selective, biology-driven approaches should be considered. Prospective studies are needed for validation.

**Supplementary Information:**

The online version contains supplementary material available at 10.1245/s10434-026-19192-1.

Intrahepatic cholangiocarcinoma (iCCA), perihilar cholangiocarcinoma (pCCA), and gallbladder carcinoma (GBC) are aggressive biliary tract malignancies for which radical resection remains the only potentially curative treatment. Lymphadenectomy (LND), the removal and pathological assessment of regional lymph nodes, is recommended as part of curative-intent surgery in biliary tract cancers (BTC), although its implementation in routine practice and its therapeutic value remain controversial.^1–3^ Lymph node metastases (LNM) affect approximately 30–45% of patients with iCCA, 40–60% of patients with pCCA and 50–70% of patients with GBC.^4–10^ The presence and extent of LNM are strong prognostic factors in all three tumor types and associated with poor survival. Current guidelines therefore recommend retrieving a minimum of six lymph nodes to optimize staging accuracy and better predict outcomes.^4,10–12^

However, the extent and technique of LND vary significantly across institutions, and adherence to these recommendations remains inconsistent.^12–14^ Although several studies suggest that systematic LND may improve staging and potentially impact survival, others question its benefit, particularly in node-negative disease, and point to increased surgical risks. LND has been associated with longer operation times, increased intraoperative blood loss with higher transfusion rates, and a greater incidence of surgical complications.^1,7,15^ Overall morbidity rates following radical resection of iCCA, pCCA, and GBC range between 25 and 42%, reflecting surgical complexity.^4,7,16^ Additionally, recurrence remains a significant concern, often linked to microscopic tumor spread.^8,11^ Tumor-related factors such as large size, multifocality, positive margins, and advanced T stage may also interact with nodal status to influence prognosis.^8,9,15^

The therapeutic value of LND in biliary tract cancers remains controversial. Unlike most prior studies, our retrospective cohort uniquely stratified patients with iCCA, pCCA, and GBC into no, limited (1–5 nodes), and extended (≥ 6 nodes) LND, allowing for simultaneous evaluation of postoperative morbidity and survival outcomes.

## Patients and Methods

### Study Design and Study Cohort

All patients with a diagnosis of iCCA, pCCA and GBC undergoing hepatic resection at the Department for Visceral, Thoracic, and Vascular Surgery at the University Hospital Dresden between 1 January 2013 and 1 April 2023 were prospectively collected in an electronic database.^17^ The cohort was retrospectively analyzed according to the extent of LND.

### Definition of Lymphadenectomy (Lymph Node Dissection)

LND was defined as the removal of one or more groups of lymph nodes during primary tumor resection. Limited LND was assumed when less than six lymph nodes were present in the resection specimen according to the evaluating pathologist (LND1–5). LND was classified as guideline-concordant according to the American Joint Committee on Cancer (AJCC) standards, referring to the removal of six or more lymph nodes (LND ≥ 6).^10,11,18^

### Primary and Secondary Outcome Analysis

Primarily, this study aimed to elucidate the influence of limited versus extended LND on major morbidity, classified as Clavien–Dindo classification grade ≥ III. Secondly, the effects of LND on the occurrence of surgical and medical complications, operation time, reoperation, the length of the intensive care unit (ICU) and overall hospital stay, perioperative transfusion rate, RFS, and OS were assessed. The last follow-up of all patients was performed on 25 October 2023.

### Ethicala Approval

This study was conducted according to the Declaration of Helsinki with waivers of consent of all patients. Ethical approval of the study protocol was obtained by the local ethics committee before analysis (approval no.: BO-EK-342082025).

### Statistical Analysis

Analyses were performed using SPSS v29.0.2.0 (IBM Corp.). Continuous variables were reported as mean ± standard deviation (SD) or median [interquartile range (IQR)] and compared with *t*-test, Mann–Whitney *U*, or Kruskal–Wallis tests as appropriate. Categorical variables were compared with chi-squared or Fisher’s exact test. For three-group comparisons, analysis of variance (ANOVA) was used. All tests were two-sided, with *p* ≤ 0.05 considered significant; Bonferroni correction was applied for subgroup comparisons (*p* ≤ 0.0167). Logistic regression was used to identify predictors of postoperative morbidity, expressed as odds ratios (OR). Variables with *p* < 0.10 in univariable analysis were entered into multivariable stepwise models. Cox regression was used for recurrence-free survival (RFS) and overall survival (OS), with hazard ratios (HR) reported.

## Results

### Characteristics of the Lymph Node Dissection (LND)

LND was performed in 47.0% of the overall cohort (*n* = 253), with extended yield (≥ 6 nodes) in 52.9% of these cases. The frequency and extent of LND varied by tumor type. GBC had the highest LND rate (64.0%), followed by pCCA (54.9%) and iCCA (40.8%). The proportion with ≥ 6 nodes removed was similar in pCCA (56.4%) and GBC (62.5%), but lower in iCCA (48.4%). Median LN counts reflected these differences (6 for GBC, 7 for pCCA, and 4.5 for iCCA; Table 1).

**Table 1:** Description of lymphadenectomy for the overall cohort and each group of biliary tract cancers

	Total cohort	iCCA	pCCA	GBC
*Overall*	*n* = 253	*n* = 157	*n* = 71	*n* = 25
Excised LNDs, *n* [%]				
0	134 [53.0]	93 [59.2]	32 [45.1]	9 [36.0]
≥ 1*	119 [47.0]	64 [40.8]	39 [54.9]	16 [64.0]
1–5	56 [47.1]	33 [51.6]	17 [43.6]	6 [37.5]
≥ 6	63 [52.9]	31 [48.4]	22 [56.4]	10 [62.5]
*LND^+^ subgroup	*n* = 119	*n* = 64	*n* = 39	*n* = 16
Tumor-positive, *n* [%]	45 [37.8]	21 [32.8]	18 [46.2]	6 [28.6]
Number of excised LND(s) (median [IQR])	6 [3–12]	4.5 [2.3–11.0]	7 [4–12]	6 [3.5–15.8]
*Major resections*	*n* = 161	*n* = 89	*n* = 62	*n* = 10
Excised LNDs, *n* [%]				
0	70 (43.5)	42 (47.2)	26 (41.9)	2 (20)
≥ 1	91 (56.5)	47 (52.8)	36 (58.1)	8 (80)
1–5	43 (26.7)	25 (28.1)	16 (25.8)	2 (20)
≥ 6	48 (29.8)	22 (24.7)	20 (32.3)	6 (60)
*Minor resections*	*n* = 92	*n* = 68	*n* = 9	*n* = 15
Excised LNDs, *n* [%]				
0	64 (69.6)	51 (75.0)	6 (66.7)	7 (46.7)
≥ 1	28 (30.4)	17 (25.0)	3 (33.3)	8 (53.3)
1–5	13 (14.1)	8 (11.8)	1 (11.1)	4 (26.7)
≥ 6	15 (16.3)	9 (13.2)	2 (22.2)	4 (26.7)

^*^LND ≥1 equals LND^+^ subgroup.

*CCA* cholangiocarcinoma, *iCCA* intrahepatic CCA, *pCCA* perihilar CCA, *GBC* gallbladder carcinoma, *LN* lymph node

Nodal positivity was most frequent in pCCA (46.2%), followed by iCCA (32.8%) and GBC (28.6%). Major resections, defined as the resection of three or more liver segments, were generally associated with higher rates of LND.

Among major resections, LND was performed in 80.0% of GBC, 58.1% of pCCA, and 52.8% of iCCA; among minor resections, rates were 53.3%, 33.3%, and 25.0%, respectively (Table 1).

### Clinical Characteristics and Surgical Procedure

Of the 253 patients, LND was omitted in 53.0%, limited in 22.1%, and extended in 24.9%. The mean (SD) lymph node yield was 2.7 (1.3) in the LND1–5 group and 13.4 (7.0) in the LND ≥ 6 group (*p* < 0.001; Table 2). Baseline characteristics were largely comparable, though patients with LND ≥ 6 had lower body mass index (BMI) and less diabetes. Tumor type distribution differed, with iCCA more frequent in the no LND (LND−) group and GBC in the LND ≥ 6 group (Table 2).

**Table 2:** Baseline characteristics and details of the surgical procedure of patients who underwent liver resection for intrahepatic or perihilar cholangiocarcinoma or gallbladder carcinoma stratified by lymphadenectomy

Parameters	Overall	LND^−^	LND^+^		*p-*value			
			LND^1–5^	LND^≥6^	LND^−^/ LND^1–5^/LND^≥6^	Pairwise comparisons^1^		
						LND^-^ versus LND^1–5^	LND^−^versus LND^≥6^	LND^–-5^ versus LND^≥6^
Patients, *n* (%)	253 (100)	134 (53.0)	56 (22.1)	63 (24.9)				
Number of excised LND(s) [mean (SD)]	3.9 (6.6)	0 (0)	2.7 (1.3)	13.4 (7.0)	< 0.001	< 0.001	< 0.001	< 0.001
Female gender, *n* (%)	104 (41.1)	46 (34.3)	28 (50.0)	30 (47.6)	0.065	–	–	–
Age, years [median (IQR)]	68 (60–75)	67.5 (61–74)	69 (58.5–75)	68 (59–76)	0.942	–	–	–
Body mass index, kg/m^2^ [median (IQR)]	25.9 (23.1-30.1)	27.4 (23.9–30.7)	25.6 (23.4–30.4)	24.5 (22.4–27.4)	**0.001**	0.179	**< 0.001**	0.069
ASA classification, *n* (%)								
I/II	91 (36.0)	46 (34.3)	19 (34.0)	26 (41.3)	0.599	–	–	–
III/IV	159 (62.9)	87 (64.9)	35 (62.5)	37 (58.7)	0.702	–	–	–
Diagnosis, *n* (%)								
iCCA	157 (62.1)	93 (69.4)	33 (58.9)	31 (49.2)	**0.021**	0.358	**0.007**	0.180
pCCA	71 (28.1)	32 (23.9)	17 (30.4)	22 (34.9)	0.250	–	**–**	–
GBC	25 (9.9)	9 (6.7)	6 (10.7)	10 (15.9)	0.129	–	**–**	–
Neoadjuvant systemic therapy, *n* (%)	27 (10.7)	17 (12.7)	3 (5.4)	7 (11.1)	0.347	–	–	–
Preoperative intervention								
PVE	35 (12.3)	19 (14.2)	4 (7.2)	12 (19.1)	0.173	–	–	–
ERCP with stenting	17 (6.7)	7 (5.2)	2 (3.6)	8 (12.7)	0.111	–	–	–
PTCD	11 (4.3)	2 (1.5)	4 (7.1)	5 (7.9)	0.053	–	–	–
Preexisting disorders, *n* (%)								
Diabetes	77 (30.4)	49 (36.6)	17 (30.4)	11 (17.5)	**0.025**	0.504	0.008	0.130
Chronic renal failure	42 (16.6)	23 (17.2)	10 (17.9)	9 (14.3)	0.878	–	–	–
Liver cirrhosis Child A	11 (4.3)	8 (6.0)	2 (3.6)	1 (1.6)	0.307	–	–	–
Substance use, *n* (%)								
Alcohol	16 (6.3)	9 (6.7)	4 (7.1)	3 (4.8)	0.887	–	–	–
Nicotine	35 (13.8)	22 (16.4)	7 (12.5)	6 (9.5)	0.415	–	–	–
Open surgical approach versus minimal invasive, *n* (%)	239 (94.5)	125 (93.3)	53 (94.6)	63 (100)	0.122	–	–	–
Duration of surgery, min [median (IQR)]	324 (211–430)	292 (191–393)	323 (213–425)	416 (305–506)	**< 0.001**	0.165	**< 0.001**	0.017
Major resection, *n* (%)	161 (63.6)	70 (52.2)	43 (76.8)	48 (76.2)	**< 0.001**	**0.002**	**<** **0.001**	0.827
Hepatectomy								
Right	40 (15.8)	18 (13.4)	11 (19.6)	11 (17.5)	0.531	**–**	**–**	**–**
Left	33 (13.0)	13 (9.7)	10 (17.9)	10 (15.9)	0.232	**–**	**–**	**–**
Extended hepatectomy								
Right	40 (15.8)	17 (12.7)	7 (12.5)	16 (25.4)	**0.044**	1.000	0.024	0.068
Left	22 (8.7)	8 (6.0)	9 (16.1)	5 (7.9)	0.098	**–**	**–**	**–**
Mesohepatectomy	6 (2.4)	2 (1.5)	4 (7.1)	2 (3.2)	**0.033**	0.063	1.000	0.050
ALPPS	27 (10.7)	16 (11.9)	3 (5.4)	8 (12.7)	0.330	**–**	–	–
Resection volume, cm^3^ [median (IQR)]	2805 (1248–20,475)	2520 (867–23,571)	2421 (1467–12,976)	3822 (1523–20,580)	0.608			
Vascular resection, *n* (%) Vena cava Portal vein Hepatic artery	21 (8.3) 30 (11.9) 7 (2.8)	5 (3.8) 7 (5.2) 1 (0.7)	5 (8.9) 8 (14.3) 2 (3.6)	11 (17.5) 15 (23.8) 4 (6.3)	**0.011** **< 0.001** 0.108	0.260 0.072 –	**0.003** **< 0.001** **–**	0.192 0.246 –
Biliodigestive anastomosis, *n* (%)	109 (43.1)	45 (33.6)	23 (41.1)	41 (65.1)	**< 0.001**	0.407	**< 0.001**	**0.010**
Simultaneous surgery	62 (24.5)	25 (18.7)	16 (28.6)	21 (33.3)	0.060	–	–	–
Pathology, *n* (%) pT ≥ 3 pN ≥ 1 pM1 R ≥ 1 G ≥ 2 pV_ mikro/makro_ pPn1 pL1	58 (22.9) 71 (28.0) 86 (34.0) 36 (14.2) 196 (77.4) 60 (23.7) 108 (42.7) 64 (25.3)	23 (26.6) 24 (17.9) 34 (25.4) 17 (12.6) 87 (64.9) 25 (18.6) 42 (31.3) 21 (15.7)	15 (26.7) 17 (30.4) 24 (42.9) 6 (10.7) 53 (94.6) 18 (32.2) 23 (41.1) 21 (37.5)	20 (21.7) 30 (47.6) 28 (44.4) 13 (20.6) 56 (88.9) 17 (27.0) 43 (68.3) (22 34.9)	0.056 **< 0.001** **0.003** 0.261 **< 0.001** 0.550 **< 0.001** **0.010**	– 0.081 0.028 – **< 0.001** – 0.737 **0.013**	– **< 0.001** **0.002** – **< 0.001** – **< 0.001** 0.017	– 0.062 0.561 – 0.331 – **0.003** 0.850
Adjuvant therapy Radioimmunotherapy Chemotherapy: Capecitabine Cisplatin/gemcitabine Other	41 (16.2) 1 (0.4) 24 (9.5) 11 (4.3) 5 (2.0)	16 (11.9) – 9 (6.7) 5 (3.7) 2 (1.4)	8 (14.3) – 3 (5.3) 4 (7.1) 1 (1.8)	17 (27.0) 1 (1.6) 12 (19.0) 2 (3.2) 2 (3.2)	**0.025** 0.470 0.051	0.811 – –	**0.013** **–** **–**	0.116 – –
Preoperative blood values [median (IQR)] Hemoglobin Bilirubin Albumin Platelets (GPt/L)	8.0 (7.0–8.8) 10.1 (6.5–18.8) 41.4 (34.1–45.0) 260 (196–344)	8.0 (7.2-8.9) 9.9 (6.8–17.0) 41.7 (36.0–44.8) 237 (182–326)	8.2 (7.1–8.8) 7.6 (4.8–13.4) 42.6 (34.3–45.8) 281 (198–337)	7.5 (6.6–8.5) 15.5 (7.8–41) 37.2 (29.5–44.6) 286 (231–392)	**0.023** **< 0.001** 0.075 **0.007**	0.778 0.046 – 0.159	**0.007** **0.005** **–** **0.002**	0.046 **< 0.001** **–** 0.165

All tests were two-sided, with *p* ≤ 0.05 considered significant; Bonferroni correction was applied for subgroup comparisons (*p* ≤ 0.0167)

^1^Significance level of ≤ 0.0167 due to Holm–Bonferroni correction.

*LND*^*−*^ no lymph node dissection, *LND*^*+*^ lymph node dissection, *LND*^*1–5*^ limited LND of 1 to 5 lymph nodes, *LND*^*≥6*^ extended LND of six or more lymph nodes

Operative characteristics reflected increasing complexity with LND extent. LND ≥ 6 was associated with longer operative time, more frequent major resections, vascular resection, and biliodigestive anastomosis (BDA). Surgical approach did not differ, with open surgery predominant across all groups (Table 2).

Pathology showed higher nodal positivity (pN ≥ 1), advanced tumor stage (pT ≥ 3), and more perineural (pPn1) and lymphovascular invasion (pL1) in the LND ≥ 6 group, who were also more likely to receive adjuvant therapy, particularly chemotherapy.

### Postoperative Parameters and Complications

#### Overall Cohort

Major complications occurred in 49.8% overall, with higher rates after LND ≥ 6 (69.8%) compared with LND− (41.8%) and LND1–5 (46.4%; *p* < 0.001; Table 3). Minor complications (24.5%) and reoperation (23.7%) did not differ significantly between groups. Among surgical events, bilioma was more frequent with LND ≥ 6 (28.6%, *p* = 0.004), while bile leakage grade B occurred more often in LND1–5 (19.6%, *p* = 0.007). Other surgical complications, including wound problems, liver failure, bleeding, and BDA insufficiency, were not associated with LND extent.

**Table 3:** Outcome analysis for patients who underwent liver resection for intrahepatic or perihilar cholangiocarcinoma or gallbladder carcinoma stratified by lymphadenectomy

	Total cohort	LND^−^	LND^+^		p-value			
			LND^1-5^	LND^≥6^	LND^−^/LND^1–5^/LND^≥6^	Pairwise comparisons		
						LND^−^ versus LND^1–5^	LND^−^ versus LND^**≥6**^	LND^1–5^ versus LND^**≥6**^
Patients, *n* (%)	253 (100)	134 (53.0)	56 (22.1)	63 (24.9)				
Postoperative complications Clavien–Dindo I–II Clavien–Dindo ≥ III	62 (24.5) 126 (49.8)	31 (23.1) 56 (41.8)	16 (28.6) 26 (46.4)	15 (23.8) 44 (69.8)	0.758 **< 0.001**	0.630	**< 0.001**	**0.015**
Surgical complications, *n* (%) Bilioma Bile leakage grade A B C Insufficient BDA Wound healing insufficiency Postoperative bleeding Liver failure Grad A B C	39 (15.4) 9 (3.6) 29 (11.5) 12 (4.7) 31 (12.3) 51 (20.2) 22 (8.7) 7 (2.8) 19 (7.5) 2 (0.8)	15 (11.2) 9 (6.7) 17 (12.7) 8 (6.0) 12 (9.0) 22 (16.4) 9 (6.7) 4 (3.0) 7 (5.2) 2 (1.5)	6 (10.7) 0 (–) 11 (19.6) 3 (5.4) 7 (12.5) 14 (25.0) 5 (8.9) 2 (3.6) 7 (12.5) 0 (–)	18 (28.6) 0 (–) 1 (1.6) 1 (1.6) 12 (19.0) 15 (23.8) 8 (12.7) 1 (1.6) 5 (7.9) 0 (–)	**0.004** **0.018** **0.007** 0.451 0.137 0.300 0.384 0.884 0.215 0.884	1.000 **0.018** 0.262 – - - – – – –	**0.004** 0.060 **0.014** – - - – – – –	**0.021** 0.060 **0.001** – - - – – – –
Medical complications Overall [mean ± SD] Delirium, *n* (%) Urinary infection, *n* (%) Vein thrombosis, *n* (%) Lung embolism Pneumonia, *n* (%) Pleural effusion, *n* (%) Intermittent dialysis, *n* (%) Sepsis, *n* (%) CPR, *n* (%)	1.2 ± 1.6 12 (4.7) 9 (3.6) 12 (4.7) 22 (8.7) 43 (17.0) 44 (17.4) 13 (5.1) 27 (10.7) 12 (4.7)	1.1 ± 1.6 6 (4.5) 5 (3.7) 6 (4.5) 10 (7.5) 16 (11.9) 16 (11.9) 6 (4.5) 12 (9.0) 8 (6.0)	1.0 ± 1.6 2 (3.6) 1 (1.8) 1 (1.8) 6 (11.3) 9 (17.0) 9 (17.0) 4 (7.1) 3 (5.4) 2 (3.6)	1.4 ± 1.4 4 (6.3) 3 (4.8) 5 (7.9) 6 (9.5) 18 (28.6) 19 (30.2) 3 (4.8) 12 (19.0) 2 (3.2)	**0.018** 0.791 0.746 0.303 0.797 **0.014** **0.006** 0.695 **0.034** 0.731	0.769 – – – – 0.483 0.483 – 0.559 –	**0.010** – – – – **0.005** **0.003** – 0.060 –	**0.016** – – – – 0.127 0.085 – 0.029 –
Re-surgery, *n* (%)	60 (23.7)	28 (21.0)	17 (30.4)	15 (23.8)	0.393			
Perioperative transfusions [mean ± SD] Packed red blood cells Platelets FFPs	1.2 ± 4.2 0.4 ± 1.5 2.0 ± 5.9	± 4.3 0.4 ± 1.6 2.0 ± 5.6	1.4 ± 4.5 0.3 ± 1.3 1.5 ± 4.3	1.1 ± 4.0 0.4 ± 1.7 2.5 ± 7.3	0.751 0.801 0.987	**–** **–** **–**	– – –	– – –
Days in the ICU [median (IQR)]	3 (1–11)	2 (0.8–11)	3 (1–7)	5 (2–18)	**0.006**	0.737	**0.002**	0.021
Days in the hospital [median (IQR)]	20 (11–38)	15 (9–28)	19 (11–41)	35 (19–53)	**< 0.001**	0.078	**< 0.001**	**0.008**

All tests were two-sided, with *p* ≤ 0.05 considered significant; Bonferroni correction was applied for subgroup comparisons (*p* ≤ 0.0167)

*LND*^*−*^ no lymph node dissection, *LND*^*+*^ lymph node dissection, *LND*^*1–5*^ limited LND of 1 to 5 lymph nodes, *LND*^*≥6*^ extended LND of six or more lymph nodes

Medical morbidity increased with LND extent (*p* = 0.018). LND ≥ 6 was associated with higher rates of pneumonia (28.6%, *p* = 0.014), pleural effusion (30.2%, *p* = 0.006), and sepsis (19.0%, *p* = 0.034), while other medical events were not significantly different.

Median ICU and hospital stay rose with LND extent (*p* = 0.006 and *p* < 0.001, respectively, Table 3). Transfusion requirements were similar.

#### Subgroup Analyses

In the subgroup of major resections (*n* = 161), major complications occurred in 64.6%, with highest rates in LND ≥ 6 (83.3%) compared with LND− (58.6%) and LND1–5 (53.5%; *p* = 0.004; Supplementary Table 1). Bilioma was more frequent with LND ≥ 6 (37.5%, *p* = 0.002), while bile leakage grade B was more common in LND1–5 (20.9%, *p* = 0.045). Other surgical and medical events were similar. ICU stay was longer in LND ≥ 6 (median 9 versus 6 days; *p* = 0.035), and hospital stay prolonged (37 days; *p* < 0.001).

### Risk Factors for Major Postoperative Complications

#### Overall Cohort (*n* = 253)

In univariable analysis, limited LND was not associated with increased risk (OR 1.207, 95% CI 0.645–2.261, *p* = 0.556), whereas extended LND was linked to higher odds of major complications (OR 1.796, 95% CI 1.305–2.471, *p* < 0.001, Supplementary Table 2). When directly compared with limited LND, the risk with LND ≥ 6 remained significantly higher (OR 3.050, 95% CI 1.658–5.613, *p* < 0.001; Supplementary Table 2). However, in multivariable regression, LND ≥ 6 versus LND0–5 did not retain independent significance (OR 2.076, 95% CI 0.898–4.797, *p* = 0.087; Supplementary Table 2). Instead, other perioperative factors emerged as predictors: BDA (OR 4.559, 95% CI 1.792–11.602, *p* = 0.001), major versus minor resection (OR 3.4399, 95% CI 1.469–7.863, *p* = 0.006), positive resection margin (R ≥ 1; OR 3.480, 95% CI 1.132–10.704, *p* = 0.029), and intraoperative packed red blood cell (PRBC) transfusion (OR 1.439, 95% CI 1.046–1.980, *p* = 0.024; Supplementary Table 2). Other variables, including age, sex, ASA status, vascular reconstruction, or minimally invasive approach, were not independently associated with higher morbidity.

#### Subgroup Analyses

Among major resections (*n* = 161), multivariable analysis identified LND ≥ 6 as an independent risk factor for major complications (OR 2.792, 95% CI 1.121–6.955, *p* = 0.027), in addition to BDA (OR 4.075, *p* = 0.001). Transfusion showed a trend toward significance (OR 1.229, *p* = 0.070), while other surgical or preoperative variables were not associated (Supplementary Table 3).

In minor resections (*n* = 92), LND was not linked to increased morbidity in univariable analysis, and the limited number of events (*n* = 22) precluded multivariable testing (Supplementary Table 4).

In the subgroup of major resections for iCCA (*n* = 89), LND ≥ 6 was a strong independent predictor of major complications (OR 7.258, 95% CI 1.630–32.314, *p* = 0.009), together with BDA (OR 8.645, 95% CI 2.191–34.109, *p* = 0.002) and diabetes (OR 6.018, 96% CI 1.782–20.323, *p* = 0.004; Supplementary Table 5).

### Risk Factors for Recurrence

Cox regression analyses were performed in 212 patients, 13 patients were lost to follow-up, and 28 patients died within 30 days after surgery. The median follow-up time for the total study population was 267 days (IQR 93–636 days) for RFS (Table 4). In univariable analysis, LND ≥ 6 was associated with increased recurrence risk compared with no LND (HR 1.674, 95% CI 1.048–2.673, *p* = 0.031) and LND1–5 (HR 1.557, 95% CI 1.012–2.393, *p* = 0.044), but this effect was not retained in multivariable models (HR 0.786, 95% CI 0.382–1.619, *p* = 0.513). Neoadjuvant chemotherapy remained independently associated with recurrence (HR 2.06, 95% CI 1.01–4.17, *p* = 0.046). Lower BMI and advanced pathological stage (pT ≥ 3), nodal involvement, and distant metastasis were significant in univariable analysis, but only pT ≥ 3 remained independently predictive (HR 2.01, 95% CI 1.10–3.19, *p* = 0.021).

**Table 4:** Prediction of postoperative tumor recurrence in uni- and multivariate regression analysis [n = 212, exclusion of 30-day-mortality (n = 28) and lost to follow-up (n = 13)].

	Univariate Cox regression			Multivariate Cox regression		
	HR	95% CI	*p*-Value	HR	95% CI	*p*-Value
Lymphadenectomy *n* ≥ 1 versus *n* = 0 *n* = 1–5 versus *n* = 0 *n* ≥ 6 versus *n* = 0 *n* ≥ 6 versus *n* = 1–5 *n* ≥ 6 versus *n* = 0–5	1.431 1.226 1.674 1.404 1.557	0.960–2.132 0.753–1.998 1.048–2.673 0.829–2.378 1.012–2.393	0.078 0.413 **0.031** 0.207 **0.044**	– – 0.786 – –	– – 0.382–1.619 – –	– – 0.513 – –
Female gender Age BMI ASA ≥ 3 Diagnosis GBC pCCA iCCA **Neoadjuvant therapy versus upfront** Preexisting Diabetes Liver cirrhosis	1.165 0.998 0.941 0.851 1.716 0.809 0.918 2.165 0.874 0.840	0.780–1.740 0.980–1.017 0.902–0.981 0.568–1.275 0.974–3.023 0.490–1.334 0.605–1.395 1.259–3.722 0.569–1.343 0.367–1.920	0.456 0.867 **0.004** 0.434 **0.062** 0.406 0.690 **0.005** 0.540 0.679	– – 0.971 – 1.406 – – 2.056 – –	– – 0.915–1.030 – 0.623–3.171 – – 1.013–4.173 – –	– – 0.333 – 0.412 – – **0.046** – –
Major resection Vascular reconstruction Simultaneous extrahepatic resection	1.237 2.283 1.052	0.824–1.859 1.398–3.726 0.648–1.708	0.305 **< 0.001** 0.837	– 1.229 –	– 0.575–2.628 –	– 0.595 –
**Overall complication (yes versus no)** Minor complications Major complications (Clavien ≥ 3) Re-surgery Duration of hospitalization	1.528 0.881 1.250 1.542 1.007	1.010–2.310 0.560–1.388 0.839–1.863 0.919–2.587 1.000–1.014	**0.045** 0.586 0.272 0.101 **0.058**	1.820 – – – 1.000	0.972–3.405 – – – 0.988–1.012	0.061 – – – 0.951
Pathology, *n* (%) ** pT ≥ 3** pN ≥ 1 pM1 R ≥ 1 G ≥ 2 pV_ mikro/makro_ pPn1 pL1	2.477 2.111 1.859 1.495 1.067 1.553 0.067 1.782	1.588–3.864 1.354–3.290 1.153–2.997 0.812–2.754 0.652–1.744 0.986–2.445 0.974–2.232 1.148–2.764	**< 0.001** **< 0.001** **0.011** 0.197 0.797 **0.057** **0.067** **0.010**	2.013 1.748 0.811 – – 1.163 1.098 1.386	1.102–3.189 0.628–5.410 0.260–2.128 – – 0.830–2.155 0.582–2.072 0.714–2.691	**0.021** 0.266 0.581 – – 0.232 0.773 0.335
Perioperative transfusions (scale) PRBC Plasma	1.021 0.984	0.905–1.152 0.907–1.068	0.737 0.702	– –	– –	**–** **–**
Adjuvant therapy	1.417	0.900–2.231	0.133	–	–	**–**

Variables with *p* < 0.10 in univariable analysis were entered into multivariable stepwise models

**Fig. 1:**
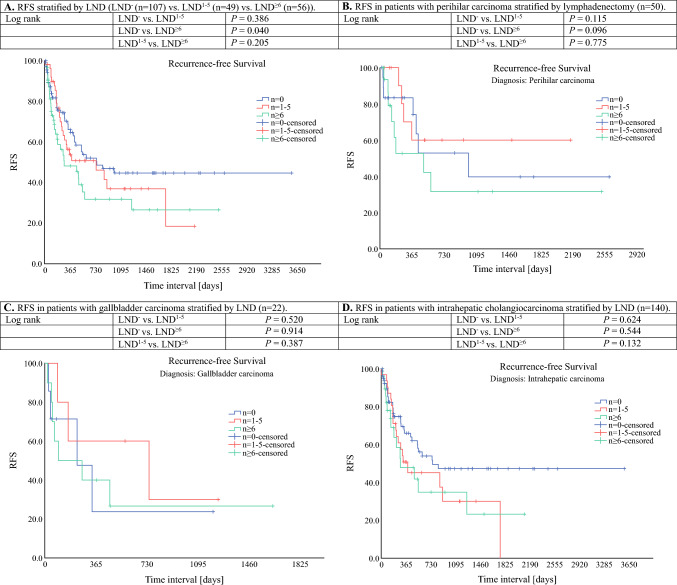
Kaplan–Meier curves for recurrence-free survival (RFS); **A** RFS stratified by LND (LND−, *n* = 107 vs. LND1–5, *n* = 49 vs. LND ≥ 6, *n* = 56); **B** RFS in patients with perihilar carcinoma (pCCA) stratified by lymphadenectomy (*n* = 50); **C** RFS in patients with gallbladder carcinoma (GBC) stratified by LND (*n* = 22); **D** RFS in patients with intrahepatic cholangiocarcinoma (iCCA) stratified by LND (*n* = 140)

RFS stratified by tumor type and LND extent is shown in Fig. 1. In iCCA, patients with LND ≥ 6 had significantly worse RFS than those with no or limited LND (log-rank *p* = 0.043). In pCCA, RFS was lower with LND ≥ 6 but not significant (*p* = 0.106). No differences were observed for GBC (*p* = 0.582).

### Risk Factors for Overall Survival (OS)

Cox regression analysis for OS was performed in 225 patients; 28 patients who died within 30 days after surgery were excluded. The median follow-up time for the total study population was 384 days (IQR 130–1018 days) for OS (Table 5).

**Table 5:** Prediction of postoperative overall survival in uni- and multivariate Cox regression analysis (n = 225, exclusion of 30-day mortality (n = 28)

	Univariable Cox regression			Multivariable Cox regression		
	HR	95% CI	*p*-Value	HR	95% CI	*p*-Value
Lymphadenectomy						
*n* ≥ 1 versus *n* = 0	1.176	0.832–1.663	0.358	–	–	–
*n* = 1–5 versus *n* = 0	1.062	0.688–1.640	0.785	–	–	–
*n* ≥ 6 versus *n* = 0	1.143	0.928–1.409	0.209	–	–	–
*n* ≥ 6 versus *n* = 1–5	1.286	0.786–2.104	0.317	–	–	–
*n* ≥ 6 versus *n* = 0–5	1.291	0.872–1.913	0.202	–	–	–
Female gender	0.971	0.679–1.388	0.872	–	–	–
Age	1.020	1.002–1.038	**0.030**	1.016	0.996–1.036	0.127
BMI	0.970	0.938–1.004	**0.080**	1.011	0.968–1.056	0.632
ASA ≥ 3	1.460	1.006–2.117	**0.046**	1.247	0.794–1.959	0.338
Diagnosis						
GBC	0.858	0.462–1.592	0.627	–	–	–
pCCA	1.383	0.940–2.036	0.100	–	–	–
iCCA	0.811	0.567–1.161	0.252	–	–	–
Neoadjuvant therapy versus upfront	1.785	1.096–2.910	**0.020**	1.869	0.995-3.509	*0.052*
Preexisting						
Diabetes	1.040	0.726–1.491	0.830	–	–	–
Liver cirrhosis	1.576	1.024–2.425	**0.039**	1.262	0.507–2.997	0.644
Major resection	1.551	1.077–2.234	**0.018**	1.152	0.701–1.893	0.576
Vascular reconstruction	2.211	1.441–3.390	**< 0.001**	1.451	0.844–2.495	0.178
Simultaneous extrahepatic resection	1.272	0.821–1.972	0.282	–	–	–
Overall complication (Y/N)	1.509	1.055–2.159	**0.024**	–	–	–
Major complications (Clavien ≥ 3)	2.030	1.436–2.870	**< 0.001**	1.410	0.892–2.230	0.142
Duration of hospitalization	1.017	1.012–1.022	**< 0.001**	1.008	1.000–1.015	*0.056*
Pathology, *n* (%)						
pT ≥ 3	2.129	1.429–3.172	**< 0.001**	1.646	1.000–2.709	*0.050*
pN ≥ 1	2.582	1.753–3.803	**< 0.001**	0.449	0.057–3.560	0.448
pM1	2.839	1.915–4.209	**< 0.001**	4.929	0.635–38.261	0.127
R ≥ 1	2.485	1.480–4.174	**< 0.001**	1.793	0.953–3.374	*0.070*
G ≥ 2	1.081	0.704–1.660	0.723	–	–	–
pV_mikro/makro_	1.801	1.165–2.784	**0.008**	1.324	0.816–2.148	0.255
pPn1	1.870	1.300–2.690	**< 0.001**	1.158	0.727–1.845	0.537
pL1	1.745	1.177–2.586	**0.006**	0.973	0.583–1.626	0.918
Perioperative transfusions (yes/no)						
PRBC	1.803	1.197–2.714	**0.005**	1.110	0.652–1.087	0.700
Plasma	1.062	1.024–1.102	**0.001**	1.035	0.985–1.087	0.171
Adjuvant therapy	0.653	0.396–1.075	**0.093**	0.763	0.412–1.413	0.389
Tumor recurrence	1.313	0.919–1.877	0.135	–	–	–

In the uni- and multivariable Cox regression analysis of OS, LND was not identified as a significant predictor. Borderline independent predictors of worse OS included neoadjuvant therapy (HR 1.869, *p* = 0.052), duration of hospitalization (HR 1.008, *p* = 0.056), pT ≥ 3 (HR 1.646, *p* = 0.050), and R ≥ 1 (HR 1.793, *p *= 0.070). Other factors were not independently associated with survival.

## Discussion

In this retrospective cohort study of 253 patients undergoing oncologic resection for iCCA, pCCA, and GBC, we assessed the impact of LND on postoperative morbidity, RFS and OS.

In our cohort, LND was performed in fewer than half of all patients (47%), and LND ≥ 6 was observed in just over half of those cases (52.9%). Our findings resemble the variability in LND practices previously reported.^1,4,12,14^ These findings could be attributed to nonadherence to guideline recommendations or the beginning of participant inclusion in 2013. Consistent with prior findings, we observed a higher frequency of LND in patients with pCCA and GBC compared to iCCA, reflecting differences by tumor type.^3,11^ Importantly, the stratified design of our study (LND– versus LND1–5 versus LND ≥ 6) allowed us to distinguish between no, limited, and extended LND, providing a more detailed assessment.

We observed that extent of LND was associated with increased postoperative morbidity. Major complications occurred in 69.8% of patients with LND ≥ 6, compared with 41.8% in LND– and 46.4% in LND1–5 (*p* < 0.001). However, this association did not remain significant in the overall cohort after multivariable adjustment and was instead confined to specific subgroups, most notably patients undergoing major liver resection and the iCCA major-resection subgroup. These findings are consistent with earlier studies and meta-analyses indicating that extended LND increases operation time, technical complexity of the surgical procedure, intraoperative blood loss and perioperative risk.^7,15,16,19,20^ The need for BDA, intraoperative PRBC transfusions, vascular reconstruction, and major hepatectomy, each more common in the LND ≥ 6 group, also contributed significantly to morbidity, in line with the current literature.^16,17,21^

From an oncological perspective, the therapeutic benefit of LND remains questionable, and our results should be interpreted with caution given the retrospective design, absence of standardized criteria for LND, and size of the cohort. In our study, recurrence occurred within the first year of follow-up with a median of 267 days. Although univariable analysis showed worse RFS with LND ≥ 6, multivariable models did not confirm this finding. In tumor-specific Kaplan–Meier analysis, the iCCA subgroup exhibited a borderline significant difference in RFS with extensive LND (log-rank *p* = 0.043), although this result should be interpreted with caution due to the above outlined limitations. No significant differences were found for pCCA or GBC. Additionally, multivariable Cox regression confirmed that the extent of LND was not independently associated with OS. These findings resonate with the results of a recent meta-analysis by Li et al.^22^ in which no significant difference in RFS and OS in iCCA with or without LND was observed. Importantly, our study stratified outcomes by nodal status. Among patients with node-negative disease, neither RFS nor OS significantly differed between those with guideline-concordant and limited LND. However, these findings should be interpreted carefully. Our findings are consistent with the established principle that, while LND improves staging accuracy, it is unlikely to influence disease trajectory in patients without nodal metastases.^4,12,22^ Although our data do not allow us to draw firm conclusions about the value of extended LND, prior studies have highlighted its role in accurate staging.^6,23^ In our cohort, pN ≥ 1 demonstrated an association with RFS and OS in univariate analyses; however, these findings were not sustained in multivariate models. This loss of significance could be explained by several limitations, including small and heterogeneous subgroups, limited statistical power, potential confounding by other pathological features, and the selection bias inherent to the retrospective design. Notably, our results highlighted other critical predictors of poor outcomes, including pT ≥ 3, R1 resection status, and duration of hospitalization, all of which influenced survival. These observations suggest that biologically driven surgical approaches may be more relevant to long-term outcomes than the extent of LND itself. Our findings do not allow us to determine whether the retrieval of ≥ 6 lymph nodes has a direct therapeutic impact, although this threshold has been recommended to improve staging accuracy and guide adjuvant treatment decisions.^24,25^ The absence of a survival benefit with more extensive LND calls for further refinement in patient selection. Advanced age above 60 years, increased tumor size of > 5 cm, and lymph node-positive disease were independent prognostic risk factors for OS in iCCA.^5^ In parallel, enhanced imaging models combined with clinical data may help predict node-positive disease.^26^

Our findings offer new perspectives to the existing literature. Comparison of no LND with limited and extended LND, rarely addressed in past studies, allowed us to highlight the difference in the adequacy of LND but also the uncertainty surrounding the use of limited LND. Limited LND was not associated with differences in RFS or OS compared with no LND. Although consistent with previous work suggesting that limited LND is unlikely to provide a survival benefit, these findings remain exploratory.^12^ Given the lack of prognostic clarity and the absence of a morbidity difference compared with no LND, the clinical value of limited LND remains uncertain.

The simultaneous analysis of iCCA, pCCA, and GBC, provides a real-world overview of surgical outcomes across biliary tract cancers. Subgroup analysis of iCCA, pCCA, and GBC revealed no clear differences in RFS or OS associated with varying degrees of LND. These exploratory observations could suggest that the prognostic effect of LND is limited across these tumor types. In addition to confirming previously described associations between guideline-concordant LND (≥ 6) and higher rates of major complications, our study contributes new perspectives on underreported medical complications.^24^ We observed a significantly increased rate of pleural effusion in the LND ≥ 6 group (28.6%) compared with both LND– (11.9%) and LND1–5 (17.0%) patients. While complications such as bile leakage and liver failure have been consistently associated with extensive dissection in biliary tract surgery, pleural effusion has received considerably less attention in prior studies. This may reflect a broader systemic response to prolonged operation times, increased manipulation near the diaphragm, or fluid shifts associated with larger resections and LND. These findings highlight the importance of considering the full spectrum of surgical and medical morbidity when determining the extent of LND.

Our exploratory findings support the growing recognition that the primary value of LND in BTC lies in its contribution to accurate staging rather than direct therapeutic gain. This concept is reinforced by previous studies, which demonstrated that the survival benefit of LND in GBC was limited and highly dependent on factors such as nodal involvement and baseline tumor biology.^27^ Similarly, in the context of iCCA, recent evidence suggests that while LND improves nodal stratification, it does not independently confer a survival benefit in node-negative patients.^4,22^ These insights are increasingly important as BTC management evolves toward more personalized and biology-driven strategies. The rise of molecular profiling and targeted therapies further shifts the emphasis from standard surgical interventions toward more tailored approaches, where the indication for LND might be guided by its diagnostic yield rather than its presumed therapeutic value.^28^

This study is subject to several limitations. First, its retrospective design carries an inherent risk of selection bias, particularly as the indication and extent of LND were based on individual surgeon discretion rather than a standardized protocol. Second, while the sample size is relatively large for this rare group of malignancies, subgroup analyses, especially for GBC, are likely underpowered and may not detect modest survival effects. Third, follow-up duration may not be sufficient to capture late recurrences, particularly in node-negative or low-risk patients. Fourth, surgeon-specific factors, such as operative technique, experience, and experience to perform more extensive lymphadenectomy, were not recorded and therefore could not be accounted for in the multivariable analysis, representing an additional source of potential confounding. Lastly, the qualitative aspects of LND, e.g., dissected nodal stations, were not assessed, which may vary across procedures and surgeons.

In conclusion, this study provides an exploratory evaluation of LND in BTC, uniquely stratifying patients into no, limited, and extended LND. While extended LND improved staging accuracy, we did not identify clear benefits in RFS or OS, but observed higher rates of major morbidity, particularly after major hepatectomy and in iCCA. These observations raise questions about the universal application of systematic LND in current guidelines and may suggest that its value could be primarily diagnostic rather than therapeutic. A more selective, biology-driven approach may help balance staging yield against operative risk; however, this requires validation in prospective multicenter studies.

## Supplementary Information

Below is the link to the electronic supplementary material.

Supplementary file1 (DOCX 36 KB)

## Data Availability

The data supporting the findings of this study are available upon reasonable request from the corresponding author.
