# Supplementary material for: Is Extended Lymphadenectomy in Biliary Tract Cancers Justified? A Retrospective Comparative Study of Gallbladder Cancer, Perihilar and Intrahepatic Cholangiocarcinoma
Source: Ann Surg Oncol. 2026 Feb 16;33(8):7010–22. doi: 10.1245/s10434-026-19192-1 (PMC13337787; doi:10.1245/s10434-026-19192-1)
Supplement: Supplementary file 1 — Supplementary file1 (DOCX 36 KB) [file 10434_2026_19192_MOESM1_ESM.docx]

**Supplementary table 1.** Outcome analysis for patients who underwent **major** liver resection for intrahepatic or perihilar cholangiocarcinoma or gallbladder carcinoma stratified by lymphadenectomy.

LND^-^ – no lymph node dissection; LND^+^ – lymph node dissection; LND^1-5^ – limited LND of 1 to 5 lymph nodes; LND^≥6^ – extended LND of six or more lymph nodes.

|  | **Total cohort** | **LND^-^** | **LND^+^** | | *p*-value | | | |
| --- | --- | --- | --- | --- | --- | --- | --- | --- |
|  |  |  | **LND^1-5^** | **LND^≥6^** | LND^-^ / LND^1-5^ / LND^≥6^ | *Pairwise comparisons* | | |
|  |  |  |  |  |  | *LND^-^ / LND^1-5^* | *LND^-^ / LND***^≥6^** | *LND1^-5^ / LND***^≥6^** |
| Patients [n (%)] | 161 (100) | 70 (43.5) | 43 (26.7) | 48 (29.8) |  |  |  |  |
| Complications [n (%)]   - Clavien-Dindo I-II - Clavien-Dindo ≥III | 34 (21.1)  104 (64.6) | 14 (20.0)  41 (58.6) | 13 (30.2)  23 (53.5) | 7 (14.6)  40 (83.3) | 0.181  **0.004** | -  0.369 | **-**  **0.005** | **-**  **0.003** |
| Surgical complications [n (%)]   - Bilioma - Bile leakage Grade   - A   - B   - C - Insufficient biliodigestive anastomosis - Wound healing insufficiency - Postoperative bleeding - Liver failure Grad   - A   - B   - C | 34 (21.1)  5 (3.1)  16 (9.9)  6 (3.7)  28 (17.4)  41 (25.5)  21 (13.0)  5 (3.1)  14 (8.7)  5 (3.1) | 12 (17.1)  5 (7.1)  6 (8.6)  3 (4.3)  10 (14.3)  15 (21.4)  8 (11.4)  3 (4.3)  3 (4.3)  3 (4.3) | 4 (9.3)  0 (-)  9 (20.9)  2 (4.7)  7 (16.3)  13 (30.2)  5 (11.6)  1 (2.3)  6 (14.0)  1 (2.3) | 18 (37.5)  0 (-)  1 (2.1)  1 (2.1)  11 (22.9)  13 (27.1)  8 (16.7)  1 (2.1)  5 (10.4)  1 (2.1) | **0.002**  **0.045**  **0.010**  0.768  0.451  0.575  0.697  0.738  0.177  0.738 | 0.190  0.155  0.086  -  -  -  -  -  -  - | 0.018  0.079  0.238  -  -  -  -  -  -  - | **0.003**  **-**  **0.006**  -  -  -  -  -  -  - |
| Medical complications   - Overall [mean±SD] - Delirium [n (%)] - Urinary infection [n (%)] - Vein thrombosis [n (%)] - Lung embolism - Pneumonia [n (%)] - Pleural effusion [n (%)] - Intermittent dialysis [n (%)] - Sepsis [n (%)] - CPR [n (%)] | 1.5 ±1.6  12 (7.5)  5 (3.1)  9 (5.6)  17 (10.6)  36 (22.4)  36 (22.4)  12 (7.5)  27 (16.8)  11 (6.8) | 1.6 ±1.4  6 (8.6)  2 (2.9)  4 (5.7)  8 (11.4)  11 (15.7)  11 (15.7)  5 (7.1)  12 (17.1)  7 (10.0) | 1.2 ±1.6  2 (4.7)  1 (2.3)  1 (2.3)  4 (9.3)  9 (20.9)  9 (20.9)  4 (9.3)  3 (7.0)  2 (4.7) | 1.6 ±1.5  4 (8.3)  2 (4.2)  9 (18.8)  5 (10.4)  16 (33.3)  16 (33.3)  3 (6.3)  12 (25.0)  2 (4.2) | 0.243  0.801  1.000  0.563  0.949  *0.079*  *0.079*  0.863  *0.075*  0.484 | **-**  **-**  **-**  **-**  **-**  **-**  **-**  **-**  **-** | **-**  **-**  **-**  **-**  **-**  **-**  **-**  **-**  **-** | **-**  **-**  **-**  **-**  **-**  **-**  **-**  **-**  **-** |
| Re-Surgery [n (%)] | 50 (31.1) | 19 (27.1) | 16 (37.2) | 15 (31.3) | 0.527 | - | - | - |
| Perioperative transfusions [mean ± SD]   - Packed Red blood cells - platelets - FFPs | 1.7 ± 5.2  0.6 ± 1.9  3.0 ± 7.1 | 2.0 ± 5.8  0.8 ± 2.1  3.6 ± 7.4 | 1.7 ± 5.1  0.4 ± 1.5  1.7 ± 4.9 | 1.3 ± 4.6  0.6 ± 1.9  3.3 ± 8.3 | 0.916  0.302  0.329 | -  -  - | **-**  **-**  **-** | **-**  **-**  **-** |
| Days in the ICU [median (IQR)] | 5 (2-16) | 6 (2-16) | 3 (2-9) | 9 (3-25) | **0.035** | 0.081 | 0.286 | **0.010** |
| Days in the hospital [median (IQR)] | 27 (14-45) | 18 (13-35) | 24 (13-45) | 37 (22-63) | **<0.001** | 0.219 | **<0.001** | **0.012** |

^1^significance level of ≤0.0167 due to Holm-Bonferroni correction

**Supplementary table 2.** Prediction of major postoperative complications classified as Clavien-Dindo ≥III in uni- and multivariate regression analysis (n=253).

|  | Univariable logistic regression | | | Multivariable logistic regression | | |
| --- | --- | --- | --- | --- | --- | --- |
|  | OR | 95%CI | p-value | OR | 95%CI | p-value |
| Lymphadenectomy   - *n=1-5 vs. n=0* - *n*≥*6 vs. n=0* - *n*≥6 *vs. n=0-5* | 1.207  1.796  3.050 | 0.645-2.261  1.305-2.471  1.658-5.613 | 0.556  **<0.001**  **<0.001** | -  -  2.076 | -  -  0.898-4.797 | -  -  0.087 |
| Female gender  Age  BMI  ASA ≥III  Diagnosis   - iCCA - pCCA - GBC   Neoadj.Therapy  Preoperative Intervention  Preexisting   - Diabetes - Renal failure - Liver cirrhosis   Alcohol abuse  History of liver surgery | 1.155  1.014  0.924  1.210  0.377  3.638  0.772  0.786  2.524  0.839  2.306  0.283  0.585  0.411 | 0.700-1.907  0.992-1.037  0.876-0.974  0.726-2.017  0.223-0.638  2.006-6.599  0.336-1.773  0.352-1.754  1.389-4.585  0.491-1.435  1.149-4.627  0.076-1.054  0.206-1.661  0.163-1.038 | 0.573  0.216  **0.003**  0.464  **<0.001**  **<0.001**  0.542  0.556  **0.002**  0.521  **0.019**  **0.060**  0.314  **0.060** | -  -  0.962  -  1.965  1.560  -  -  0.781  -  2.236  0.700  -  0.492 | -  -  0.894-1.035  -  0.515-7.499  0.362-6.718  -  -  0.311-1.958  -  0.870-5.744  0.129-3.787  -  0.136-1.776 | -  -  0.301  -  0.305  0.537  -  -  0.604  -  0.098  0.667  -  0.276 |
| Minimal invasive vs. open  Duration of surgery  **Major vs. minor resection**  Resection volume  **BDA**  Vascular reconstruction  Simultaneous extrahepatic resection | 0.084  1.006  5.995  1.000  6.508  5.451  1.853 | 0.011-0.663  1.004-1.008  3.338-10.767  1.000-1.000  3.728-11.366  2.502-11.875  1.033-3.326 | **0.019**  **<0.001**  **<0.001**  0.776  **<0.001**  **<0.001**  **0.039** | 0.803  1.000  3.399  -  4.559  1.268  1.631 | 0.086-7.541  0.997-1.004  1.469-7.863  -  1.792-11.602  0.448-3.588  0.716-3.713 | 0.847  0.839  **0.006**  **-**  **0.001**  0.685  0.252 |
| Pathology [n (%)]   - G≥2 - **R≥1** | 1.492  5.190 | 0.822-2.707  2.170-12.416 | 0.188  **<0.001** | -  3.480 | -  1.132-10.704 | -  **0.029** |
| Intraoperative transfusions of   - **PRBC** - Plasma | 1.495  1.146 | 1.244-1.797  1.048-1.253 | **<0.001**  **0.003** | 1.439  0.889 | 1.046-1.980  0.779-1.014 | **0.024**  0.076 |

**Supplementary table 3.** Prediction of major postoperative complications classified as Clavien-Dindo ≥III (n=104) in uni- and multivariate regression analysis in the **subgroup of major resections** (n=161).

|  | Univariable logistic regression | | | Multivariable logistic regression | | |
| --- | --- | --- | --- | --- | --- | --- |
|  | OR | 95%CI | p-value | OR | 95%CI | p-value |
| Lymphadenectomy   - n=1-5 vs. n=0 - n≥6 vs. n=0 - n≥6 vs. n=0-5 | 0.813  3.537  3.828 | 0.379-1.748  1.444-8.662  1.644-8.915 | 0.597  **0.006**  **0.002** | -  -  2.792 | -  -  1.121-6.955 | -  -  **0.027** |
| Female gender  Age  BMI  ASA ≥III  Neoadj.Therapy  Preoperative Intervention  Preexisting   - Diabetes - Renal failure - Liver cirrhosis   History of liver surgery | 1.357  0.999  0.950  1.653  0.677  2.344  1.077  1.458  0.802  0.516 | 0.695-2.648  0.969-1.029  0.882-1.024  0.854-3.203  0.238-1.925  1.108-4.959  0.511-2.270  0.597-3.560  0.130-4.947  0.171-1.554 | 0.371  0.945  0.180  0.136  0.464  **0.026**  0.845  0.407  0.812  0.239 | -  -  -  -  -  1.085  -  -  -  - | -  -  -  -  -  0.452-2.604  -  -  -  - | -  -  -  -  -  0.855  -  -  -  - |
| Minimal invasive vs. open  Duration of surgery  Resection volume  BDA  Vascular reconstruction  Simultaneous extrahepatic resection | -  1.004  1.000  4.867  3.243  1.372 | -  1.001-1.006  1.000-1.000  2.427-9.758  1.387-7.583  0.648-2.905 | -  **0.004**  0.609  **<0.001**  **0.007**  0.408 | 0.999  -  4.075  1.770  - | 0.996-1.002  -  1.745-9.513  0.659-4.752  - | 0.554  -  **0.001**  0.257  - |
| Intraoperative transfusions of   - PRBC - Plasma - Platelets | 1.325  1.058  1.669 | 1.085-1.616  0.975-1.148  0.687-4.052 | **0.006**  0.173  0.258 | 1.229  -  - | 0.983-1.537  -  - | 0.070  -  - |

**Supplementary table 4.** Prediction of major postoperative complications classified as Clavien-Dindo ≥III (n=22) in uni- and multivariate regression analysis in the **subgroup of minor resections** (n=92).

|  | Univariable logistic regression | | | Multivariable logistic regression | | |
| --- | --- | --- | --- | --- | --- | --- |
|  | OR | 95%CI | p-value | OR | 95%CI | p-value |
| Lymphadenectomy   - n=1-5 vs. n=0 - n≥6 vs. n=0 - n≥6 vs. n=0-5 | 0.980  1.188  1.090 | 0.238-4.030  0.330-4.282  0.574-2.069 | 0.978  0.792  0.792 | -  -  - | -  -  - | **-**  **-**  **-** |

**Supplementary table 5.** Prediction of major postoperative complications classified as Clavien-Dindo ≥III in uni- and multivariate regression analysis in the **subgroup of major resections in iCCA** (n=89).

|  | Univariable logistic regression | | | Multivariable logistic regression | | |
| --- | --- | --- | --- | --- | --- | --- |
|  | OR | 95%CI | p-value | OR | 95%CI | p-value |
| Lymphadenectomy   - n=1-5 vs. n=0 - n≥6 vs. n=0 - n≥6 vs. n=0-5 | 0.951  7.667  7.811 | 0.351-2.576  1.966-29.896  2.109-28.932 | 0.921  **0.003**  **0.002** | 7.258 | 1.630-32.314 | **0.009** |
| Female gender  Age  BMI  ASA ≥III  Neoadj.Therapy  Preoperative Intervention  Preexisting   - Diabetes   History of liver surgery | 1.513  1.001  0.973  2.062  1.061  3.971  2.759  0.625 | 0.641-3.575  0.963-1.041  0.883-1.072  0.872-4.877  0.357-3.155  1.198-13.162  1.055-7.217  0.176-2.225 | 0.345  0.950  0.576  0.099  0.916  **0.024**  **0.039**  0.468 | 2.478  6.018 | 0.548-11.216  1.782-20.323 | 0.239  **0.004** |
| Duration of surgery  BDA  Vascular reconstruction  Simultaneous extrahepatic resection | 1.003  6.406  2.800  1.163 | 1.000-1.007  2.279-18.005  0.910-8.611  0.462-2.925 | **0.031**  **<0.001**  **0.072**  0.748 | 0.999  8.645  1.582 | 0.994-1.004  2.191-34.109  0.333-7.509 | 0.719  **0.002**  0.654 |
| Intraoperative transfusions of   - PRBC | 1.222 | 0.995-1.501 | **0.056** | 1.018 | 0.774-1.338 | 0.899 |
